# A spectrophotometric coupled enzyme assay to measure the activity of succinate dehydrogenase^☆^

**DOI:** 10.1016/j.ab.2013.07.018

**Published:** 2013-11-01

**Authors:** Andrew J.Y. Jones, Judy Hirst

**Affiliations:** Medical Research Council Mitochondrial Biology Unit, Cambridge CB2 0XY, UK

**Keywords:** Coupled assay, Fumarate hydratase, Oxaloacetate decarboxylating malic enzyme, Succinate:ubiquinone oxidoreductase

## Abstract

Respiratory complex II (succinate:ubiquinone oxidoreductase) connects the tricarboxylic acid cycle to the electron transport chain in mitochondria and many prokaryotes. Complex II mutations have been linked to neurodegenerative diseases and metabolic defects in cancer. However, there is no convenient stoichiometric assay for the catalytic activity of complex II. Here, we present a simple, quantitative, real-time method to detect the production of fumarate from succinate by complex II that is easy to implement and applicable to the isolated enzyme, membrane preparations, and tissue homogenates. Our assay uses fumarate hydratase to convert fumarate to malate and uses oxaloacetate decarboxylating malic dehydrogenase to convert malate to pyruvate and to convert NADP^+^ to NADPH; the NADPH is detected spectrometrically. Simple protocols for the high-yield production of the two enzymes required are described; oxaloacetate decarboxylating malic dehydrogenase is also suitable for accurate determination of the activity of fumarate hydratase. Unlike existing spectrometric assay methods for complex II that rely on artificial electron acceptors (e.g., 2,6-dichlorophenolindophenol), our coupled assay is specific and stoichiometric (1:1 for succinate oxidation to NADPH formation), so it is suitable for comprehensive analyses of the catalysis and inhibition of succinate dehydrogenase activities in samples with both simple and complex compositions.

In mammalian mitochondria, succinate:ubiquinone oxidoreductase (succinate dehydrogenase, complex II, EC 1.3.5.1) catalyzes the oxidation of succinate to fumarate in the matrix and the reduction of ubiquinone-10 in the inner membrane [1,2]. Thus, it links the tricarboxylic acid cycle to the respiratory electron transport chain. Complex II comprises four subunits [1–3]. In the hydrophilic domain, the largest subunit houses a covalently bound FAD (flavin adenine dinucleotide) cofactor and the succinate-binding site, and a smaller subunit contains three iron–sulfur clusters that transfer electrons from the flavin to ubiquinone. The binding site for the ubiquinone substrate is located close to a heme cofactor in the membrane-associated domain that is composed of two hydrophobic subunits.

Homologues of mammalian complex II are widely conserved in aerobic organisms, in both eukaryotes and prokaryotes. Some prokaryotes use fumarate as a terminal electron acceptor in anaerobic respiration by using enzymes that are closely related to complex II to catalyze the reverse reaction, reduction of fumarate to succinate [4]. Some parasites, such as *Ascaris suum*, switch between succinate oxidation and fumarate reduction during different life stages, and a similar switch has been proposed to occur in mammalian cells during hypoxia [5]. Mutations in complex II have been identified as a cause of Leigh syndrome, a neurodegenerative disease [6], and complex II was recently identified as a significant source of mitochondrial reactive oxygen species production [7]. Complex II mutations have also been linked to hereditary paragangliomas, tumors in the head and neck [8], and there is currently significant interest in how mitochondrial succinate and fumarate levels are linked to the stabilization of HIF-1α and, thus, to cell proliferation [9].

Due to its central role in metabolism and medicine, measurements of complex II activity have long been used to assess the activity of both the enzyme itself and the mitochondrial electron transport chain as a whole. However, there is no convenient and reliable method for testing complex II activity. Clark oxygen electrodes are often used [10] (see, e.g., Refs. [7,11]), but they are expensive in their sample requirements, and because they measure succinate:O_2_ oxidoreduction they can assess only the combined activity of respiratory complexes II, III, and IV. Alternative real-time assays of complex II rely on artificial electron acceptors that change color on reduction and, therefore, can be monitored spectrophotometrically. 2,6-Dichlorophenolindophenol (DCPIP)^1^ and 2-(4-iodo-phenyl)-3-(4-nitrophenyl)-5-phenyl tetrazolium chloride (INT) are reduced directly by complex II and also by the quinol product of complex II catalysis (see, e.g., Refs. [12–14]). DCPIP and other tetrazolium salts (which do not react efficiently with complex II directly) have also been used as secondary electron acceptors, with phenazine methosulfate (PMS) to mediate electron transfer from complex II (see, e.g., Refs. [12,14,15]). However, all of these assays lack specificity. When either the electron acceptor or PMS reacts with the enzyme directly it fails to focus on the complete catalytic cycle of succinate:ubiquinone oxidoreduction, the reactivity is not specific to complex II, and O_2_ interferes with the results [16]. On the other hand, the reaction with ubiquinol is inefficient, electrons are not scavenged stoichiometrically when the respiratory chain is turning over, and the measured rate is always a combination of all possible pathways.

Here, we present a convenient, stoichiometric, and precise coupled enzyme assay for succinate oxidation (see Scheme 1). Our assay uses two enzymes: fumarate hydratase (FumC, EC 4.2.1.2) converts the fumarate product of succinate oxidation to malate [17], and then oxaloacetate decarboxylating malic dehydrogenase (MaeB, EC 1.1.1.40) couples the conversion of malate to pyruvate to the reduction of NADP^+^ to NADPH [18]; the NADPH is detected spectrophotometrically. Because our assay quantifies succinate oxidation directly, it is suitable for measuring succinate:ubiquinone oxidoreduction by isolated complex II as well as succinate consumption in membrane and tissue preparations.

## Materials and methods

### Preparation of FumC and MaeB

FumC and MaeB from *Escherichia coli* were overexpressed in *E. coli.* Plasmid constructs encoding the *fumC* and *maeB* genes were supplied by Todd Weaver (University of Wisconsin–La Crosse) [17] and María Drincovich (National University of Rosario, Argentina) [18], respectively. The same protocol, adapted from Refs. [17] and [18], was used for expression and purification of both proteins unless otherwise stated. *E. coli* BL21(DE3) cells (New England Biolabs) were transformed with the *maeB* or *fumC* plasmid. Cells were grown at 32 °C in LB medium supplemented with 50 μg ml^−1^ ampicillin until *A*_600_ reached approximately 0.6, and then protein expression was induced using isopropyl β-d-1-thiogalactopyranoside (IPTG, 1 mM for FumC and 0.1 mM for MaeB) for 18 h at 20 °C. Cells were harvested by centrifugation (3500*g,* 10 min), resuspended in buffer A (50 mM potassium phosphate and 300 mM NaCl, pH 7.8, at 4 °C) for FumC or buffer B (20 mM Tris–Cl, 100 mM NaCl, 25 mM imidazole, and 10% [w/v] glycerol, pH 7.4, at 4 °C) for MaeB, and then lysed in one passage through a Constant Systems model B 2.2-kW Z cell disruptor at 30 psi. The lysates were centrifuged (150,000*g,* 45 min),the pellets were discarded, and the supernatants were filtered (0.45 μm) before being loaded onto a preequilibrated 25 ml Ni–Sepharose 6 Fast Flow column (GE Healthcare). The column was washed using buffer A + 60 mM imidazole for FumC or buffer B for MaeB, and then the required proteins were eluted in buffer A + 400 mM imidazole for FumC or buffer B + 300 mM imidazole for MaeB. Relevant fractions were identified by activity assays, pooled, and concentrated (Amicon Ultra-15 50-kDa centrifugal filter units) and then were dialyzed overnight in buffer C (10 mM Tris–SO_4_, 5 mM ethylenediaminetetraacetic acid [EDTA], and 1 mM dithiothreitol, pH 7.0, at 4 °C) for FumC or buffer D (60 mMTris–SO_4_, 20 mM β-mercaptoethanol, 25 mM imidazole, and 10% [w/v] glycerol, pH 7.4, at 4 °C) for MaeB. Typical yields were 36 and 60 mg per liter of culture for FumC and MaeB, respectively.

### Preparation of complex II-containing samples

Submitochondrial particles (SMPs) were prepared from bovine heart mitochondria as described previously [11]. Complex II was solubilized from bovine heart mitochondrial membranes (∼ 10 mg protein ml^–1^ in 10 mM Tris–SO_4_ [pH 7.4], 250 mM sucrose, 1 mM EDTA, and 0.005% phenylmethanesulfonyl fluoride [PMSF]) by the addition of 0.5% *n*-dodecyl-β-d-maltopyranoside (DDM); the sample was stirred on ice for 30 min and then centrifuged (48,000*g*, 30 min), and the supernatant was collected. Complex I was prepared from bovine heart mitochondria as described previously [19]. Tissue samples were prepared from rat skeletal muscle using a procedure modified fromRef. [20]. Briefly, the leg muscle was stripped of fat and connective tissue and chopped finely (<1 mm pieces), and then it was homogenized in 15 volumes of buffer (320 mM sucrose and 10 mM Tris–HCl, pH 7.0, at 4 °C) using a tight-fitting glass Teflon homogenizer. The sample was centrifuged at 1000*g* for 5 min at 4 °C, and the supernatant was collected.

### Kinetic activity measurements

All assays were carried out in 10 mM Tris–SO_4_ (pH 7.4 at 32 °C), 250 mM sucrose, 2 mM MgSO_4_, and 1 mM K_2_SO_4_ at 32 °C unless otherwise stated. Gramicidin (20 μg ml^−1^) was included to dissipate the proton motive force in SMPs. Standard concentrations were 5 mM succinate, 2 mM NADP^+^, 60 μg ml^−1^ FumC, and 300 μg ml^−1^ MaeB. The NADPH concentration was followed at 340 to 380 nm (*ε* = 4.81 mM^−1^ cm^−1^) using a Molecular Devices SpectraMax Plus 384plate reader. The reductions of DCPIP (100 μM, 600 nm, *ε* = 21 mM^−1^ cm^−1^, blue to colorless) [14] and INT (100 μM, 500 nm, *ε* = 19 mM^−1^ cm^−1^, colorless to red) [13] were measured in the presence and absence of 100 μM decylubiquinone, 5 mM succinate, or 100 μM NADH. When required, ubiquinol oxidation by the respiratory chain was inhibited by 400 μM NaCN, and atpenin A5 (Santa Cruz Biotechnology, Santa Cruz, CA, USA) was used to inhibit complex II and added from a concentrated stocksolution in dimethyl sulfoxide (DMSO). O_2_ consumption by SMPs was measured using a Clark electrode in a stirred 1 ml Perspex cell held at 32 °C (Rank Brothers, Cambridge, UK).

## Results and discussion

### Kinetic characterization of FumC and MaeB

**Fig.1:**
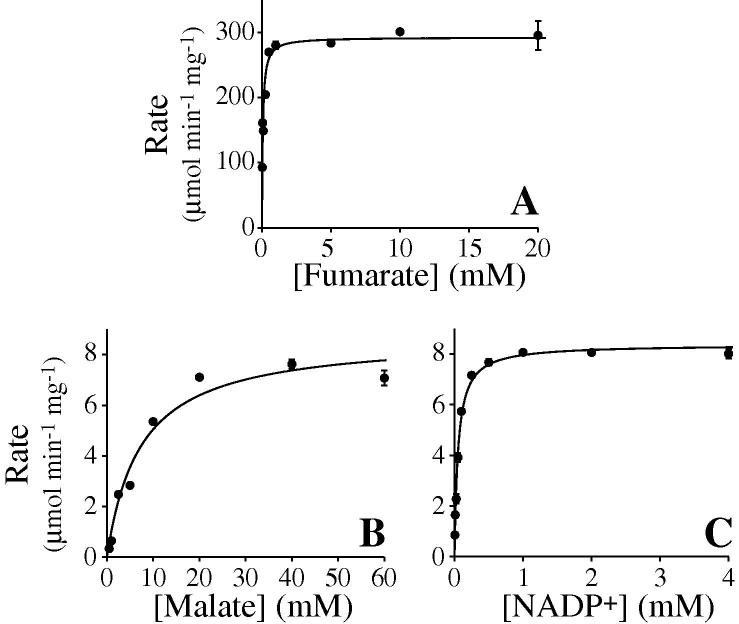
Kinetic characterization of the FumC and MaeB enzymes. (A) Rate of conversion of fumarate to malate by FumC, detected by the production of NADPH that is driven by the conversion of malate to pyruvate by MaeB. The concentrations were 1.7 μg ml^−1^ FumC, 300 μg ml^−1^ MaeB, and 2 mM NADP^+^. (B,C) Rates of conversion of malate to pyruvate by MaeB, detected by the production of NADPH. The concentrations were 44.2 μg ml^−1^ MaeB and 4 mM NADP^+^ (B) and 40 mM malate (C). In all cases, assays were carried out in triplicate with standard error bars reported, at 32 °C, in buffers containing 10 mM Tris–SO_4_ (pH 7.4), 250 mM sucrose, 2 mM MgSO_4_, and 1 mM K_2_SO_4_.

Fig. 1 shows how the rates of catalysis by the FumC and MaeB enzymes used here depend on the concentrations of fumarate and of malate and NADP^+^, respectively. First, the assay buffer used for all experiments contained 2 mM Mg^2+^ because MaeB requires Mg^2+^ for catalysis [18]; as expected, no catalysis was observed in the absence of Mg^2+^, and concentrations above 4 mM have been shown previously to inhibit [18]. K^+^ (2 mM) was also included in the assay buffer because it was shown previously to activate MaeB by approximately 30% [18]. The rate of MaeB catalysis is easily measured by the accumulation of NADPH at 340 to 380 nm; Fig. 1B shows that the apparent *K*_M_ for malate is relatively high (7.3 ± 0.9 mM) and the *V*_max_ is poor (8.37 ± 0.08 μmol min^−1^ mg^−1^ or 92 ± 0.93 s^−1^), and Fig. 1C shows that for NADP^+^ the *K*_M_ is lower (52 ± 2.6 μM). Both *K*_M_ values were measured with the second substrate at a high enough concentration for *V*_max_. Our values are comparable to those reported previously (*K*_M(malate)_ = 3.4 mM, *K*_M(NADP+)_ = 41.5 μM, *V*_max_ = 67 s^−1^) [18]. Fig. 1A shows how the rate of conversion of fumarate to pyruvate, in the presence of FumC and MaeB, depends on the concentration of fumarate present; the *K*_M_ for fumarate is 64 ± 7.8 μM and *V*_max_ is 293 ± 7 μmol min^−1^ mg^−1^ or 975 ± 21.7 s^−1^. The values are similar to those reported previously (207 μM and 1149 s^−1^) [21]. For these measurements, the concentration of MaeB was set to 300 to 600 μg ml^−1^ (0.45–0.90 μM) to overcome its poor *V*_max_ value; increasing the MaeB concentration further did not increase the observed rate of catalysis (see below also). The concentration of NADP^+^ was set to 2 mM, approximately 20 times higher than *K*_M_. Along with the release of CO_2_, the high NADP^+^/NADPH ratio precludes the reverse reaction, conversion of pyruvate to malate, which has been demonstrated to occur slowly under some conditions [18]. The lower *K*_M_ and higher *V*_max_ values observed for FumC allowed it to be used at lower concentrations, 60 to 120 μg ml^−1^ (0.3–0.6 μM), than MaeB in subsequent experiments (see below also).

Previously, the decarboxylating malic enzyme from pigeon liver (equivalent to MaeB) was used in an assay for fumarate hydratase activity [22]. Similar assays have been used recently in studies of fumarate hydratase deficiencies (see, e.g., Ref. [23]). However, commercially available decarboxylating malic enzymes (EC 1.1.1.38, -.39, or -.40) are prohibitively expensive, precluding them from being applied extensively in kinetic studies. Alternatively, malate dehydrogenases (EC 1.1.1.37 or -.82) are much cheaper, but they catalyze reversibly to reach an equilibrium position and, thus, can provide only a comparative evaluation of enzyme activities. The MaeB preparation described here compares favorably on both criteria; it catalyzes irreversibly and can be prepared easily and cheaply in large amounts.

### Measurement of rate of succinate oxidation by submitochondrial particles

**Fig.2:**
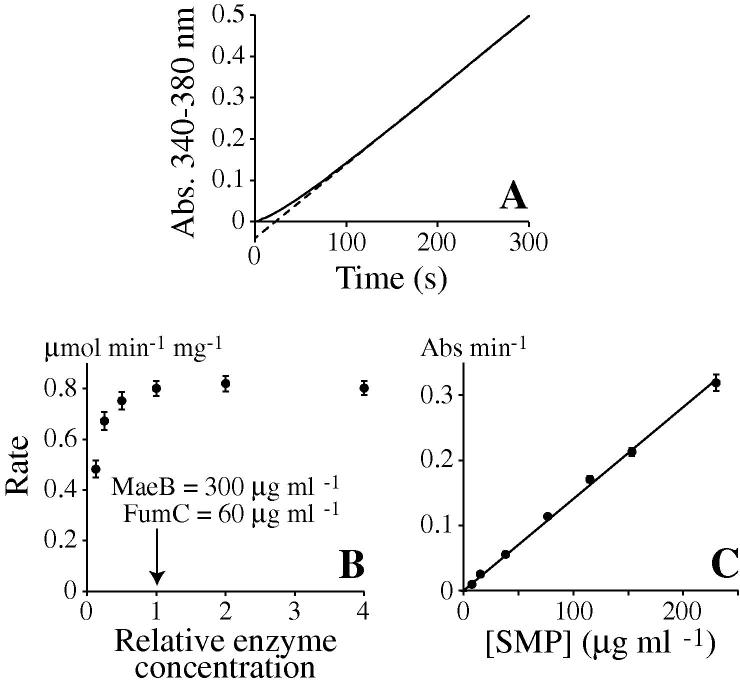
Quantifying the rate of succinate oxidation by the respiratory chain in submitochondrial particles. (A) Assay trace measuring succinate oxidation by SMPs (40 μg ml^−1^), detected by the coupled FumC–MaeB assay system as the formation of NADPH (300 μg ml^−1^ MaeB and 60 μg ml^−1^ FumC). The trace becomes linear after approximately 100 s. (B) Observed rates of succinate oxidation by SMPs (40 μg ml^−1^), detected by the coupled FumC–MaeB assay system as the formation of NADPH, over a range of FumC and MaeB concentrations. (C) Rates of succinate oxidation by SMPs, detected by the coupled FumC–MaeB assay system as the formation of NADPH, over a range of SMP concentrations (300 μg ml^−1^ MaeB and 60 μg ml^−1^ FumC). In panels B and C, assays were carried out in triplicate with standard error bars reported. In all cases, assays were at 32 °C in buffers containing 10 mM Tris–SO_4_ (pH 7.4), 250 mM sucrose, 5 mM succinate, 2 mM NADP^+^, 2 mM MgSO_4_, and 1 mM K_2_SO_4_.

Fig. 2A shows a typical assay trace recorded to follow succinate:O_2_ oxidoreduction by respiratory complexes II, III, and IV in SMPs. The rate of NADPH formation is not constant throughout the assay; it begins slowly and then speeds up to reach a constant value after approximately 100 s. During the assay “lag phase,” the intermediate fumarate and malate concentrations accumulate to their steady-state values. Thus, the lag phase can be eliminated by increasing the concentrations of FumC and MaeB. However, the final rate measurement is unchanged, and because of the increased enzyme requirements we consider this to be unnecessary.

To confirm the specificity of the coupled assay for the conversion of succinate to fumarate, rates of NADPH formation were measured in the absence of various assay components. In the absence of SMPs, succinate, MaeB, or NADP^+^ the observed rates were negligible, but in the absence of FumC a significant rate, approximately 10% of the control, was observed. Based on the other control experiments, we conclude that this results from a low level of endogenous fumarate hydratase in the SMP preparation—which does not affect the assay results because it merely reduces the load on the exogenous FumC.

Fig. 2B shows that our standard working concentrations of MaeB (300 μg ml^−1^) and FumC (60 μg ml^−1^) are adequate for the quantitative detection of succinate oxidation by SMPs (provided that the lag phases are discarded), and Fig. 2C shows that the rate of NADPH reduction by the detection system depends directly on the SMP concentration over the whole range tested. Therefore, the detection system is not rate limiting in any of these experiments, and the observed rates accurately define the rates of succinate oxidation.

### Comparison of coupled assay system with alternative methods

**Fig.3:**
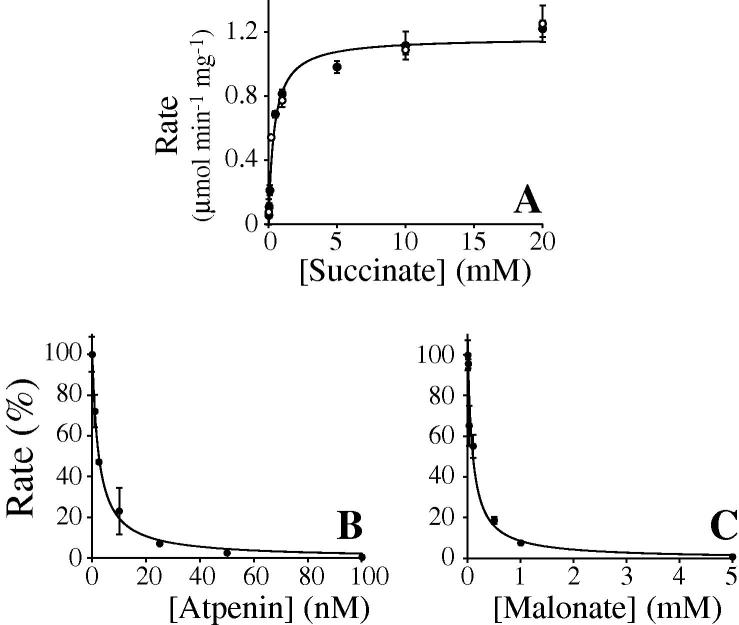
Kinetic parameters describing succinate oxidation and its inhibition by the respiratory chain in SMPs. (A) Rates of succinate oxidation by SMPs detected by the coupled assay system (solid symbols) or by the Clark O_2_ electrode (open symbols). (B,C) Rates of succinate oxidation by SMPs detected by the coupled assay in the presence of varying concentrations of atpenin A5 (B) and malonate (C). Inhibition curves were fit using the standard dose–effect relationship with Hill slope = 1. The assays contained 120 μg ml^−1^ FumC, 600 μg ml^−1^ MaeB, 5 mM succinate (B,C), 2 mM NADP^+^, 2 mM MgSO_4_, and 1 mM K_2_SO_4_ in 10 mM Tris–SO_4_ (pH 7.4) and 250 mM sucrose. The measurements were carried out in triplicate at 32 °C and are reported with standard error bars.

Succinate oxidation by complex II in SMPs is coupled to the reduction of O_2_ by complex IV, via the electron transport chain, in a 2:1 succinate:O_2_ stoichiometry. Therefore, measurements of O_2_ consumption, using a Clark electrode, are the “gold standard” for confirming the accuracy of any new assay. Fig. 3A shows that measurements of the rate of succinate oxidation by SMPs using our coupled assay system agree very well with Clark electrode measurements. Note that our assay system can be applied to measurements on isolated complex II, whereas the Clark electrode can be used only when complex II catalysis is linked to O_2_ reduction by the respiratory chain. Our assay has the additional advantages of being a spectrophotometric method, with significantly lower sample requirements, improved convenience, and quicker and easier data accumulation.

To investigate alternative methods that are applicable to isolated complex II as well as to membrane and tissue preparations, we compared our coupled assay for succinate oxidation with an assay using DCPIP [12,14] (see Table 1). The rate of succinate oxidation by detergent-solubilized bovine heart mitochondrial membranes was measured in the presence of a short-chain ubiquinone, decylubiquinone (the solubilization isolates complex II from the other enzymes and precludes significant turnover from endogenous ubiquinone-10). Using 30 μg ml^−1^ solubilized membranes, 5 mM succinate, and 100 μM decylubiquinone, the observed coupled assay rate was 0.41 ± 0.023 μmol min^−1^ mg^−1^ (the rate was negligible in the absence of any of the components). With 100 μM DCPIP, a substantial rate of DCPIP reduction (0.062 ± 0.003 μmol min^−1^ mg^−1^) was observed even in the absence of decylubiquinone. A similar result (0.088 ± 0.005 μmol min^−1^ mg^−1^) was obtained when 100 μM NADH was added instead of succinate. Therefore, DCPIP is reduced directly by the respiratory chain enzymes without the participation of ubiquinone, so the assay does not address the complete complex II turnover cycle. When 100 μM decylubiquinone and 100 μM DCPIP were present (the DCPIP should be reduced by the decylubiquinol product), the rate of DCPIP reduction increased to 0.20 ± 0.007 μmol min^−1^ mg^−1^. This value is significantly less than the value from our coupled assay even if the ubiquinone-independent rate is not subtracted. Furthermore, when the full respiratory chain is present, DCPIP is unable to compete with complex III for ubiquinol, so complex IV must be inhibited to allow ubiquinone reduction to be measured. For example, when complex I NADH:ubiquinone oxidoreduction was measured with 30 μg ml^−1^ SMPs and 100 μM NADH, in the absence of a complex IV inhibitor, the rate of NADH oxidation measured directly was 0.38 ± 0.005 μmol min^−1^ mg^−1^, but the rate of DCPIP reduction was much lower, 0.18 ± 0.009 μmol min^−1^ mg^−1^. Rates of succinate oxidation by solubilized complex II determined using 100 μM INT [13] instead of DCPIP were similarly lower than the control value (see Table 1).

### Measurement of complex II activity in tissue samples

To detect biochemical defects in complex II in patients with mitochondrial diseases, rates of complex II catalysis are typically measured in tissue homogenates from biopsy samples. Specifically, the rate of DCPIP reduction is monitored in the presence of succinate and in the presence and absence of ubiquinone-1 [24]. Therefore, measurements from our coupled assay were compared with DCPIP measurements on rat skeletal muscle homogenates (see Table 1). Our coupled assay, with 5 mM succinate, 50 μM ubiquinone-1, and 1.5 mM NaCN, gave a rate of 0.12 ± 0.006 μmol min^−1^ mg^−1^ that was fully sensitive to 2 μM atpenin A5, a complex II inhibitor [14]. Using the same substrate concentrations, the rate measured using 100 μM DCPIP was only 0.06 ± 0.001 μmol min^−1^ mg^−1^. In the absence of both ubiquinone-1 and NaCN, the rates were 0.054 ± 0.002 μmol min^−1^ mg^−1^ (coupled assay) and 0.019 ± 0.001 μmol min^−1^ mg^−1^ (DCPIP). As expected, in the absence of ubiquinone-1, NaCN inhibited succinate oxidation in the coupled assay fully, but the DCPIP assay gave a rate of 0.018 ± 0.001 μmol min^−1^ mg^−1^. Typical rates reported using succinate, ubiquinone-1, NaCN, and DCPIP in human tissue samples are 0.061 to 0.079 μmol min^−1^ mg^−1^ [24], comparable to the rate detected here in rat tissue by the same method. Thus, the same picture emerges for tissues samples as for solubilized membranes—that rates of succinate oxidation measured using DCPIP are a significant underestimate of the true rates. However, we are aware of the importance of standardized measurements for the comparison of values from tissue biopsy samples [24] and, thus, that continuing with established assay protocols may be preferable in this case.

### Determination of kinetic parameters for succinate dehydrogenase catalysis

The coupled assay was used to determine the *K*_M_ and *V*_max_ values for complex II in SMPs (see Fig. 3A) and the IC_50_ values for two complex II inhibitors (Figs. 3B and C). The *K*_M_ value obtained, 410 ± 55 μM, is within the range of values reported previously (using DCPIP) of 130 μM [25], 303 μM [26], 1.3 mM [27], and 1.5 mM [28]. By using the published value of 0.19 nmol of complex II per milligram of protein in a mitochondrial inner membrane preparation [29], our *V*_max_ of 1.2 ± 0.03 μmol min^−1^ mg^−1^ (although depending strongly on the assay conditions and composition), equates to a turnover number of 100 ± 2.7 s^−1^. Note that our value is similar to that reported previously from a Clark electrode measurement in 10 mM succinate (0.94 μmol min^–1^ mg^–1^ [30]); both values are higher than a previous value from this laboratory (0.264 μmol min^–1^ mg^–1^ [11]) due to improvements in mitochondria preparation. Fig. 3B shows that atpenin A5, an inhibitor that binds in the complex II ubiquinone-binding site, has an IC_50_ value of 2.4 ± 1.2 nM, in good agreement with the published value of 3.6 nM [14] measured using DCPIP in mitochondria. Fig. 3C shows that malonate, an inhibitor that binds in the complex II flavin site, has an IC_50_ value of 96 ± 1.3 μM, in line with the values of 42 μM in rat mitochondria [31] and 180 to 210 μM in human cell lines [32] measured using DCPIP. Inhibition of complex II by the flavin site inhibitor oxaloacetate could not be quantified because it is an intermediate of the MaeB reaction and so interferes with our detection system.

## Figures and Tables

**Scheme 1:**
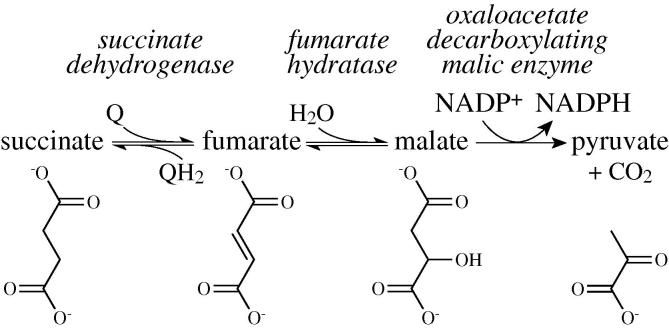
Reaction scheme for the coupled assay, illustrating the use of FumC and MaeB to couple the oxidation of succinate to the reduction of NADP^+^ in a 1:1 stoichiometry.

**Table 1:** Comparison of values measured using the different assay methods.

Preparation	Substrate(s)	Coupled assay	DCPIP	INT
Complex II^a^	Succinate + DQ	0.41 ± 0.023	0.20 ± 0.007	0.25 ± 0.024
Complex II^a^	Succinate only	0.002 ± 0.001	0.062 ± 0.003	0.036 ± 0.003
Tissue^b^ + NaCN^c^	Succinate + Q_1_	0.12 ± 0.006	0.06 ± 0.001	
Tissue^b^ + NaCN^c^	Succinate	0 ± 0.001	0.018 ± 0.001	
Tissue^b^	Succinate	0.054 ± 0.002	0.019 ± 0.001	

*Note.* All values are reported in μmol min^−1^ mg^−1^ and measured with 5 mM succinate and 100 μM decylubiquinone (DQ) or 50 μM ubiquinone-1 (Q_1_) as stated.

a Detergent-solubilized bovine heart mitochondrial membranes in which complex II is functionally isolated.

b Rat skeletal muscle homogenate.

c NaCN inhibits cytochrome *c* oxidase; it prevents reoxidation of endogenous ubiquinol-10 so that no ubiquinone-10 is present.

## Footnotes

1 *Abbreviations used:* DCPIP, 2,6-dichlorophenolindophenol; INT, 2-(4-iodo-phenyl)-3-(4-nitrophenyl)-5-phenyl tetrazolium chloride; PMS, phenazine methosulfate; FumC, fumarate hydratase; MaeB, oxaloacetate decarboxylating malic dehydrogenase; EDTA, ethylenediaminetetraacetic acid; SMP, submitochondrial particle.
